# Autopsy of a Case of Abscess of the Liver

**Published:** 1849-01

**Authors:** Halsey Rosenkrans

**Affiliations:** Ottawa, Ill.


### ARTICLE IV.

*Autopsy of a case of Abscess of the Liver.* Reported by HALSEY ROSENKRANS, M. D., of Ottawa, Ill.

On the 23d inst I was invited by Dr. Howland to witness a post mortem examination of the body of Mr. H., a man of middle age, and who had been a blacksmith by trade. I was informed that he had been sick some three or four weeks and that his illness had been considered to be asthemic Pneumonia confined to the left lung. A few day previous to his death he expectorated at times considerable quantities of very offensive sputa which was supposed by his physician to be matter from the lung in a state of gangrene. This matter, or pus, was said to have been so extremely offensive as to drive one of the Physicians (as there were two in attendance) from the room—this occurred about a week previous to his demise, when he was given up as hopeless by his attending Physicians, and Doctor Howland took charge of the case. At this time he was troubled with occasional convulsive movements and delirium, being rather furious. These paroxysms were succeeded by somnolency. This state of things continuing, if I was correctly informed, until death.

The post mortem examination was confined to the organs within the chest and the liver. Removing the sternum, we found the lungs collapsed. In attempting to raise them from their bed, we found extensive adhesions between the pleura castalis and pulmonalis. On the right side, we also found that there was extensive attachment of the lower lobe of the right lung to the diaphragm. Cutting into the substance of the lung we found the lower two lobes of the right lung and the lower lobe of the left in a state of hepatization—the upper lobe of each lung being healthy. The heart was healthy. We now proceeded to separate the attachment between the diaphragm and inferior lobe of the right lung. This being effected, pus of the most offensive character poured forth from a ragged passage through the diaphragm. Drs. Pearsons and Hopkins remarked simultaneously, that it was the same sickening smell that accompanied his sputa, more than a week previously. We next traced this opening into the right lobe of the liver and on cutting through the diaphragm into the liver, the two organs being extensively united, we found a considerable portion of the right lobe involved in the abscess. October, 1848

